# Optimal time-dependent SUC model for COVID-19 pandemic in India

**DOI:** 10.1186/s12879-024-09961-2

**Published:** 2024-09-27

**Authors:** Youngjin Hwang, Soobin Kwak, Jyoti, Junseok Kim

**Affiliations:** 1 Department of Mathematics, Korea University, Seoul, 02841 State Republic of Korea; 2 The Institute of Basic Science, Korea University, Seoul, 02841 State Republic of Korea

**Keywords:** SIR model, Time-dependent SUC model, COVID-19 pandemic, Time varying SUC model

## Abstract

In this paper, we propose a numerical algorithm to obtain the optimal epidemic parameters for a time-dependent Susceptible-Unidentified infected-Confirmed (tSUC) model. The tSUC model was developed to investigate the epidemiology of unconfirmed infection cases over an extended period. Among the epidemic parameters, the transmission rate can fluctuate significantly or remain stable due to various factors. For instance, if early intervention in an epidemic fails, the transmission rate may increase, whereas appropriate policies, including strict public health measures, can reduce the transmission rate. Therefore, we adaptively estimate the transmission rate to the given data using the linear change points of the number of new confirmed cases by the given cumulative confirmed data set, and the time-dependent transmission rate is interpolated based on the estimated transmission rates at linear change points. The proposed numerical algorithm preprocesses actual cumulative confirmed cases in India to smooth it and uses the preprocessed data to identify linear change points. Using these linear change points and the tSUC model, it finds the optimal time-dependent parameters that minimize the difference between the actual cumulative confirmed cases and the computed numerical solution in the least-squares sense. Numerical experiments demonstrate the numerical solution of the tSUC model using the optimal time-dependent parameters found by the proposed algorithm, validating the performance of the algorithm. Consequently, the proposed numerical algorithm calculates the time-dependent transmission rate for the actual cumulative confirmed cases in India, which can serve as a basis for analyzing the COVID-19 pandemic in India.

## Introduction

India, acknowledged as the world’s largest democracy, confirmed its first case of the COVID-19 pandemic on January 30, 2020. By April 21, 2024, the World Health Organization had documented a cumulative total of 45,036,197 confirmed cases and 533,581 deaths in the nation [3]. In efforts to stem the spread of the disease, both the central and state governments enforced various measures aimed at enhancing public awareness regarding COVID-19 and encouraging social distancing among citizens. A nationwide lockdown was initiated on March 24, 2020, originally intended to last 21 days but was subsequently prolonged until May 3, 2020. Despite the implementation of these measures, the viral outbreak persisted in the region due to a significant number of asymptomatic individuals and the prolonged incubation period of the virus [4].

However, the period from infection to the appearance of symptoms has an average duration of 5-6 days [5], but this range can vary significantly, extending from 1 to 14 days. Since fever is the most prevalent symptom of COVID-19 [6] and by measuring the body temperature it is identified, whether a person is infected with the virus or not. Consequently, identifying an infected person, whether asymptomatic or yet to display symptoms, becomes difficult, leading to a potential increase in the rate of COVID-19 transmission. Furthermore, a recent survey emphasized [7] that due to a significant proportion of asymptomatic individuals infected with SARS-CoV-2 in India, the reported number of cases is substantially lower than the actual number of infected individuals. Therefore, for a comprehensive understanding of the infection dynamics and population-level immunity against SARS-CoV-2 in India, accurately estimating the number of asymptomatic cases emerges as the primary concern of this study.

Several studies [8, 9] have attempted to estimate the number of asymptomatic, unidentified, or undetected COVID-19 cases in India. For instance, Saikia et al. [10] developed a Susceptible-Exposed-Infectives-Removed (SEIR) model that incorporates time-varying incubation periods and asymptomatic transmission rates. This model was used to forecast the early stages of the COVID-19 pandemic in India, based on data from eleven different states. To minimize the deviation of the solution, the authors used the derivative-free Nelder-Mead algorithm. Similarly, [11] focused on three highly impacted states in India: Maharashtra, Karnataka, and Tamil Nadu by extending the basic Susceptible-Infectious-Removed (SIR) model to incorporate ten compartments. This extension allowed for an examination of coronavirus dynamics while considering factors such as contact tracing, face mask efficacy, and the testing of quarantined and isolated individuals to estimate optimal values for disease transmission rates and the detection rates of undetected asymptomatic and symptomatic populations.

Additionally, Rakshit [12] analyzed a seven-compartment model, which includes Susceptible, Exposed, Infected, Asymptomatic, Quarantined, Fatal, and Recovered (SEIAQFR) compartments, to predict the actual number of COVID-19 cases in the UK, US, and India. The author focused on two key factors-asymptomatic transmission and patient quarantine-emphasizing that including the asymptomatic factor improved the model’s accuracy. Furthermore, [9] calculated the basic reproduction number $R_0$ for Maharashtra, India, using the Van den Driessche and Watmough methods to derive the next-generation matrix. The population was categorized into five groups: susceptible, exposed, detected-infected, undetected-infected, and recovered. They also included a separate compartment to account for the coronavirus pathogen’s presence in the environment, which affects disease transmission. However, it is important to note that their model’s estimate of unidentified case numbers was not highly accurate due to the need for approximations in some parameter values.

**Fig. 1:**
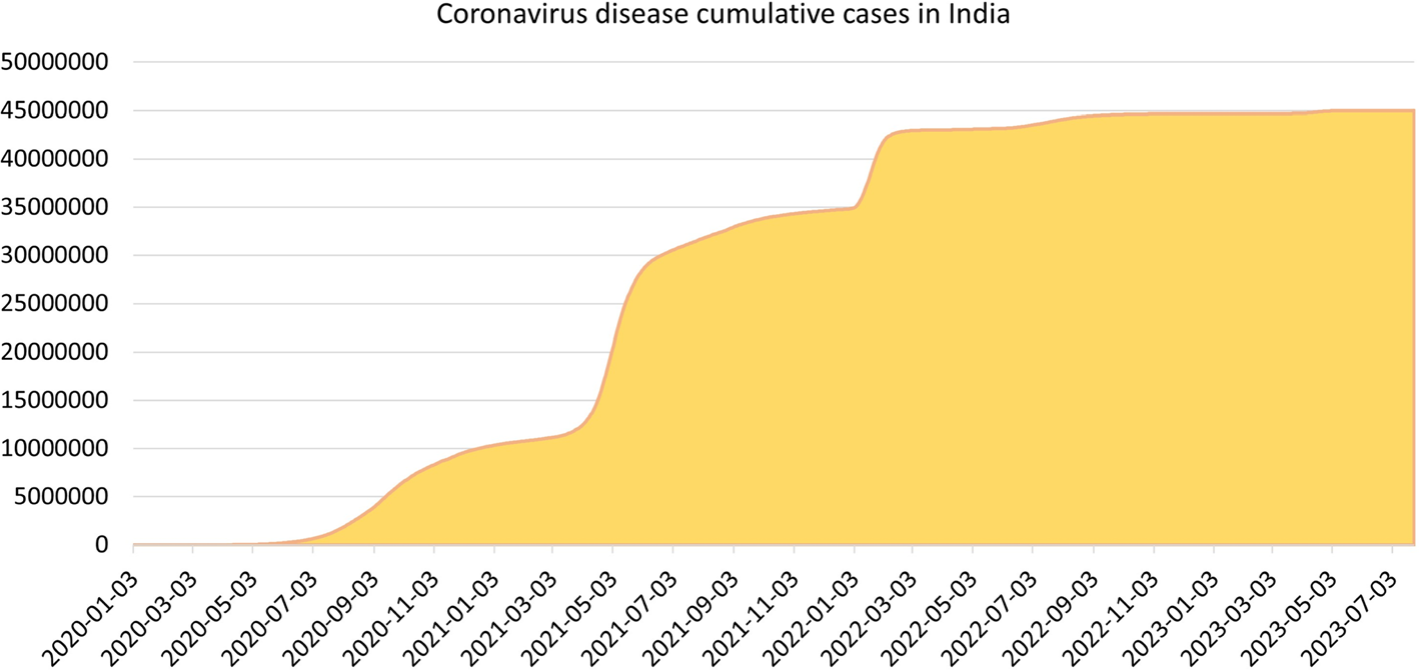
Number of population infected with COVID-19, India

Figure 1 shows the cumulative confirmed cases of COVID-19 in India from January 3, 2020 to July 26, 2023. This report was presented by the World Health Organization (WHO) [13] as of May 12, 2024 on its website. However, the reported cases may not be entirely accurate, as individuals who were asymptomatic at the time of data collection may develop symptoms later, leading to an increase in the number of reported infections over time. Hence, to present an accurate report, forecast new variants and understand the severity of the disease, it is essential to gather accurate data on unidentified or hidden infected cases in India. This information is crucial for researching transmission dynamics, risk factors, the spread magnitude, and fatality rates.

The COVID-19 pandemic has significantly challenged healthcare systems, economies, and societies across the globe. To effectively combat its impact, a deep understanding of the pandemic’s dynamics is essential. Deep learning and spectral analysis have been pivotal in modeling and predicting COVID-19 spread, demonstrating their importance in epidemiology [14–16]. The rapid transmission of the virus, particularly through variants like Delta and Omicron, highlights the urgent need for accurate forecasting models [17]. In [17], authors we leverage advanced deep learning techniques combined with spectral analysis, to predict COVID-19 future trends and uncover critical patterns, thereby providing valuable insights for public health strategies.

Recent studies [18–24] on COVID-19 epidemiology have employed various mathematical models to analyze and mitigate the pandemic impact. The susceptible-infected model is used for understanding complex behaviors and predicting the spread of COVID-19 disease [25]. Ahmed et al. [26] analyzed the COVID-19 SIR model to discuss the regional and global stability of disease-free equilibrium points and explained their biological significance. Dauji [27] analyzed the epidemiological trend in India using mathematical methods. Hajri et al. [28] presented a delayed deterministic and stochastic epidemic model to study the effects of white noise intensities. Han et al. [29] demonstrated the high potential for using machine learning methods to investigate epidemic dynamics. Lee et al. [30] discussed the construction and utilization of various infectious disease models. Lee et al. [31] developed the Susceptible-Unidentified infected-Confirmed (SUC) mathematical epidemic model for computing the unidentified infected patient.

Moreover, new infectious disease models have been developed by expanding or modifying existing ones. Meacci and Primicerio [32] proposed a Susceptible-Infected-Quarantined-Recovered-Dead (SIQRD) equation. A mathematical model was proposed to predict the exponentially decreasing case fatality rate of a pandemic within a country during its declining phase [2]. Das et al. [33] presented a multi-patch epidemic model, designed to understand how mobility influences disease spread. Their model also accounts for limited medical resources, quarantine measures, and the preventive behaviors of healthy individuals. In [34], Bandekar and Ghosh developed a 7-compartment epidemiological model that includes identified and unidentified infected populations, along with a media factor associated with the aware identified infected population. De Anda-Suarez et al. [35] developed META-COVID19, leveraging the characteristics of COVID-19 to identify the attributes of its spread across different periods of time. Zhang et al. [36] developed a framework for reliable data-driven epidemiological models, integrating data collection, model development, identifiability and sensitivity analyses, model calibration, robustness analysis, and uncertain predictions. Using this framework, they proposed a modified Susceptible-Exposed-Infectious-Recovered (SEIR) model with new compartments and vaccinations to predict COVID-19 spread in New York City. Lee et al. [37] developed a revised SUC model designed to control the COVID-19 pandemic through financial incentives. In addition, robust optimal parameters for the SUC epidemic dynamics model were derived using real-world data [38]. Hwang et al. [39] presented a time-dependent SUC (tSUC) model for a long-term analysis of the pandemic, including COVID-19.

The primary objective of this research is to investigate and analyze the epidemiology of unidentified infected individuals using the tSUC epidemiological model based on COVID-19 infected cases data in India for a long time.

The rest parts of this work are organized as follows. In Time-dependent SUC mathematical system section, we introduce the tSUC epidemic model. In Numerical solution algorithm section, we proposed the numerical algorithm. In Computational tests section, various numerical tests are performed. Conclusions are given in Conclusion section.

## Time-dependent SUC mathematical system

We consider the tSUC model [39]. The SUC model was proposed by Lee et al. [31]. Considering the conventional SIR model, *S*, *I*, and *R* represent susceptible, infected, and recovered individuals, respectively. In the context of the COVID-19 pandemic, we can further subdivide the infected group *I* into unidentified infected *U* and confirmed infected *CI*. The SUC model focuses on estimating the unidentified infected individuals responsible for disease transmission, while confirmed cases are treated as a single group that includes both isolated cases *CI*, which no longer transmit the disease, and recovered cases *R*, which also no longer transmit the disease. For more detailed information, please refer to [39]. The tSUC model considering the time-dependent transmission rate $\beta (t)$ was proposed in [39].

1 $$\begin{aligned} \frac{dS(t)}{dt} & = f\left( \beta (t),S(t),U(t)\right) = - \beta (t) \frac{ S(t) U(t)}{N}, \end{aligned}$$

2 $$\begin{aligned} \frac{dU(t)}{dt} & = g\left( \gamma ,\beta (t),S(t),U(t)\right) = \beta (t) \frac{ S(t) U(t)}{N} - \gamma U(t), \end{aligned}$$

3 $$\begin{aligned} \frac{dC(t)}{dt} & = h\left( \gamma ,U(t)\right) = \gamma U(t), \end{aligned}$$

where *S*(*t*) is the number of susceptible cases at time *t*, *U*(*t*) is the number of unidentified infected cases at time *t*, *C*(*t*) is the number of confirmed cases at time *t*, $\beta (t)$ is the transmission variable, $\gamma$ is the reciprocal of the average number of days until an unidentified infected person is confirmed, and *N* is the total population. The unidentified infected individuals transmit the disease and remain unidentified.

## Numerical solution algorithm

In this section, we present an algorithm for optimizing the time-dependent transmission rate $\beta (t)$ in the tSUC model. Let $S_n$, $U_n$, and $C_n$ be approximations of the $S(n\Delta t)$, $U(n\Delta t)$, and $C(n\Delta t)$, respectively, where $\Delta t$ is a temporal step size. Then, we can obtain the following discrete system of equations using the fourth-order Runge–Kutta (RK4) method.

4 $$\begin{aligned} S_{n+1} & = S_n + \frac{\Delta t}{6}\left( a_1+2a_2+2a_3+a_4\right) , \end{aligned}$$

5 $$\begin{aligned} U_{n+1} & = U_n + \frac{\Delta t}{6}\left( b_1+2b_2+2b_3+b_4\right) , \end{aligned}$$

6 $$\begin{aligned} C_{n+1} & = C_n + \frac{\Delta t}{6}\left( c_1+2c_2+2c_3+c_4\right) , \end{aligned}$$

where

$$\begin{aligned} a_1 & = f(\beta _n,S_n,U_n),~b_1 = g(\gamma ,\beta _n,S_n,U_n),~c_1=h(\gamma ,U_n),\\ a_2 & = f\left( \beta _{n+1/2},S_n+\frac{a_1\Delta t}{2},~U_n+\frac{b_1\Delta t}{2}\right) ,\\ b_2 & = g\left( \gamma ,\beta _{n+1/2},S_n+\frac{a_1\Delta t}{2},U_n+\frac{b_1\Delta t}{2}\right) ,\\ c_2 & = h\left( \gamma ,U_n+\frac{b_1\Delta t}{2}\right) ,\\ a_3 & = f\left( \beta _{n+1/2},S_n+\frac{a_2\Delta t}{2},U_n+\frac{b_2\Delta t}{2}\right) ,\\ b_3 & = g\left( \gamma ,\beta _{n+1/2},S_n+\frac{a_2\Delta t}{2},U_n+\frac{b_2\Delta t}{2}\right) ,\\ c_3 & = h\left( \gamma ,U_n+\frac{b_2\Delta t}{2}\right) ,\\ a_4 & = f\left( \beta _{n+1},S_n+a_3\Delta t,U_n+a_3\Delta t\right) ,\\ b_4 & = g\left( \gamma ,\beta _{n+1},S_n+a_3\Delta t,U_n+b_3\Delta t\right) ,\\ c_4 & = h\left( \gamma ,U_n+b_3\Delta t\right) , \end{aligned}$$

$\beta _n = \beta (n\Delta t)$, $\beta _{n+1/2} = 0.5(\beta (n\Delta t)+\beta ((n+1)\Delta t))$, and $U_0$ are the unknown parameters. Considering the total population *N*, $S_n=N-U_n-C_n$ must be satisfied.

### Data preprocessing

We use the following data preprocessing to generate 7-day average data from a given cumulative count of confirmed cases *C*. Let the number of given data be *M*. We calculate the count of newly confirmed cases $\Delta {\bar{C}}_m$, $m=1,2,\cdots ,M$ utilizing the provided cumulative confirmed case count ${\bar{C}}_m$, $m=0,1,\cdots ,M$.

$$\begin{aligned} \Delta {\bar{C}}_m= {\bar{C}}_m-{\bar{C}}_{m-1},~~ m=1,\cdots ,M \end{aligned}$$

We then calculate the 7-day simple moving average based on the number of new confirmed cases as follows:

$$\begin{aligned} \text {ave}\Delta {\bar{C}}_m = \frac{1}{7}\left( \Delta {\bar{C}}_{m-6}+\Delta {\bar{C}}_{m-5}+\cdots +\Delta {\bar{C}}_m\right) ,~~ m = 7,8,\cdots ,M. \end{aligned}$$

Next, we generate the smoothed cumulative confirmed cases using the 7-day simple moving average of daily new confirmed cases as

$$\begin{aligned} \text {ref}C_m = {\bar{C}}_{6}+ \sum \limits _{k=7}^m \text {ave}\Delta {\bar{C}}_k,~~m=7,8,\cdots ,M. \end{aligned}$$

**Fig. 2:**
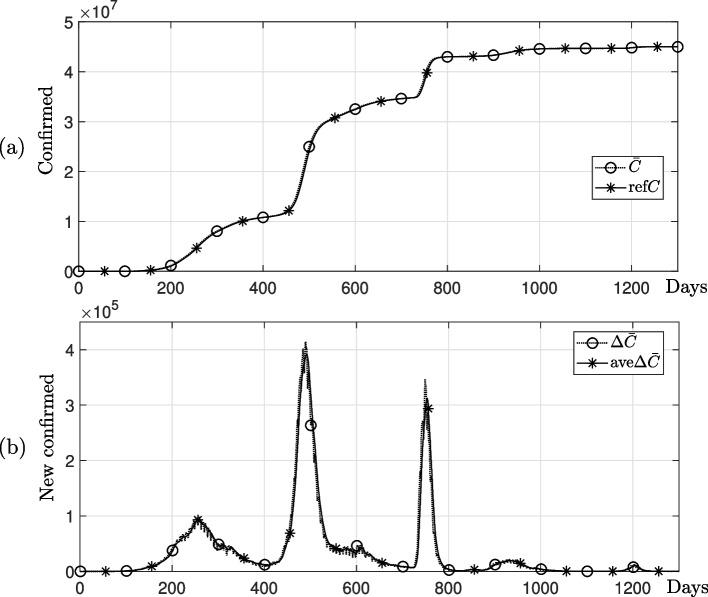
COVID-19 confirmed data in India and the smoothed confirmed data. **a** Cumulative confirmed cases. **b** New confirmed cases

Figure 2 shows the 7-day average data generated by performing data preprocessing with the number of new cases based on the data of COVID-19 cases in India.

### Estimating parameters

**Fig. 3:**
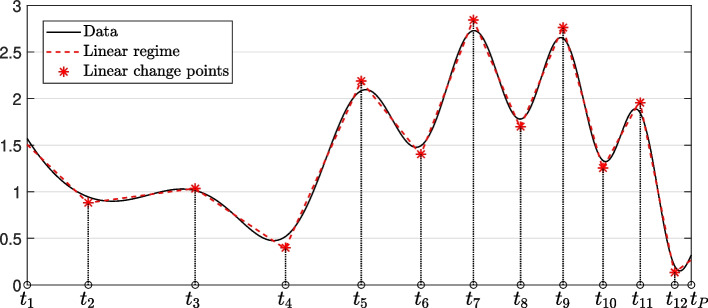
Linear change points identified using the MATLAB function ischange

When the number of new confirmed cases changes dramatically, the time-dependent transmission variable $\beta (t)$ changes together. Therefore, we determine when the time-dependent transmission variable $\beta (t)$ changes using the MATLAB function ischange, which finds the linear change point in the time series by finding the abrupt change in the slope for the calculated 7-day average data of the number of new confirmed cases. Detailed documentation on this can be found at https://uk.mathworks.com/help/matlab/ref/ischange.html, which is based on work by Killick et al. [40]. The ischange function repeatedly minimizes the cost function to determine whether the data segment has a linear change, and we use this method to identify a linear change for the number of new confirmed cases. Let *P* be a number of linear change points and let the found linear change point be $t_p \in [7,M]$, $p=2,3,\cdots ,P+1$. Here, we assume that $t_1=7< t_2<\cdots < t_{P+2}=M$. To elucidate the functionality of the MATLAB function ischange, we applied it to identify linear change points in a dataset exhibiting random variations. Figure 3 shows the linear change points found using the MATLAB ischange function with $P=11$ in the given data.

We define the sample transmission values at ${\varvec{t}}=[t_1,~t_2,~\cdots ,~t_{P+2}]$ as ${\varvec{\beta }}=[\beta _1,~\beta _2,~\cdots ,~ \beta _{P+2}]$. Then, we calculate the $\beta (t)$ at $t\in [7,M]$ by the piecewise cubic Hermite interpolating polynomial method.

To determine the optimal values of the parameters ${\varvec{\beta }}$ and $U_0$ that best align with the confirmed case data using the least-squares method.

$$\begin{aligned} \min _{{\varvec{\beta }},U_0} \frac{1}{2}\sum \limits _{m=0}^{M-7}(\text {ref}C_m-C(m)), \end{aligned}$$

where *C*(*m*) is numerical solution at the time $t=m$. We use the discrete system of Eqs. (4)–(6) and the MATLAB function lsqcurvefit [1], which is a nonlinear curve-fitting function in the least-squares sense [41].

$$\begin{aligned} [{\varvec{\beta }},~U_0] = \textbf{lsqcurvefit}('tSUCmodel',~[{\varvec{\beta }}^0,~ U_0^0],~\textbf{Tdata},~\textbf{Cdata},~lb,~ub), \end{aligned}$$

where $[{\varvec{\beta }},~U_0]$ are the estimated optimal parameters, $[{\varvec{\beta }}^0,~U_0^0]$ are the initial values, and *tSUCmodel* is a function that returns the numerical solution for the confirmed cases in the numerical solutions of the tSUC model at $\textbf{Tdata}$ by solving Eqs. 4-6. $\textbf{Cdata}$ represents a given confirmed case, we use confirmed case data in India or manufactured dataets in this paper. *lb* and *ub* are the lower and upper bounds for the parameters to be estimated, respectively.

## Computational tests

We perform the numerical experiments using the proposed numerical algorithm to find the optimal time intervals for $\beta (t)$. Then, we estimate the unidentified infected *U*(*t*) using the proposed method. We use the confirmed case data in India obtained from the data of the WHO Coronavirus dashboard from January 3, 2021, to July 26, 2023, as of July 26, 2023.

### Comparison with previous method

**Fig. 4:**
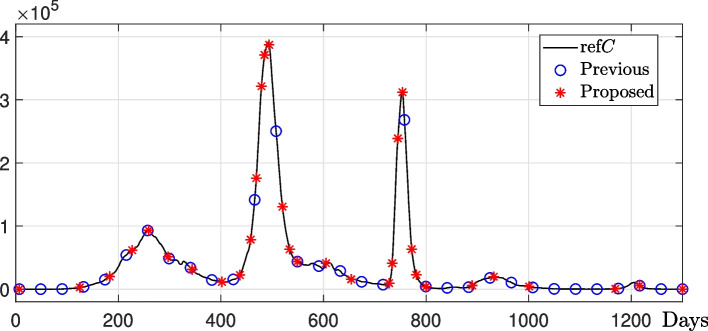
Sample points for the previous and proposed methods

We compare the proposed method with the previous method [39]. As for the previous method, sample points were defined at regular intervals, and transmission values were estimated at the sample points. Therefore, estimating transmission rates for a given data is difficult if the spacing between sample points is wide. The parameters used are the total population $N=136\times 10^7$, $\Delta t=0.1$, $\gamma =1/4$, ${\varvec{\beta }}^0=[1/3,~1/3,~\cdots ,~1/3]$, $U_0^0 = 1$, $lb = [0,~0,~\cdots ,~0,~1]$, $ub = [1,~1,~\cdots ,~1,~\infty ]$, and the number of linear change points $P=30$. Figure 4 shows the number of new confirmed cases at sample points for the previous and proposed methods. We observed that in the period where the number of new confirmed cases remains relatively steady, the previous method estimates a greater number of transmission rates than the proposed method, whereas in the period with relatively rapid changes, it estimates a smaller number of transmission rates.

**Fig. 5:**
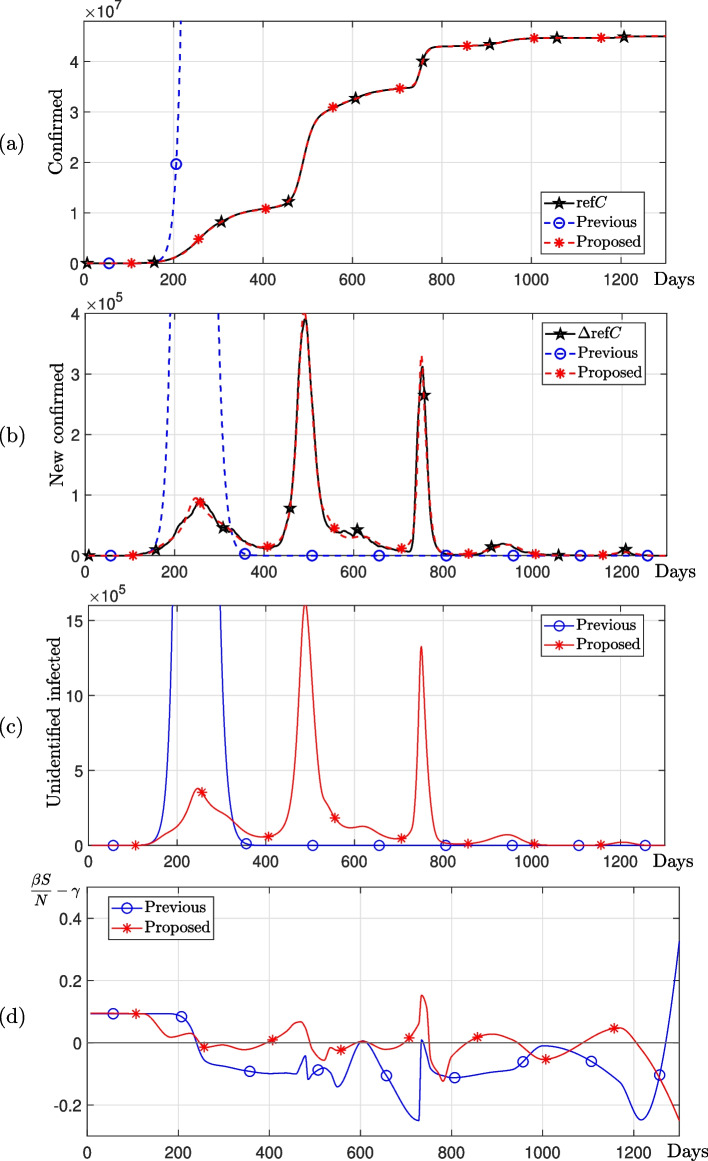
Numerical solutions using the previous and proposed methods with COVID-19 confirmed data in India. **a**–**c** are number of cumulative confirmed cases, new confirmed cases, and unidentified infected cases, respectively. **d** $\beta S/N-\gamma$

Figure 5 shows numerical solutions using the previous and proposed methods. We observed that the numerical solution calculated using the time-dependent transmission rate $\beta (t)$ estimated by the previous method does not fit the COVID-19 confirmed data in India. In contrast, the proposed method successfully estimated the optimal time-dependent transmission rate $\beta (t)$ for the COVID-19 confirmed data in India, which undergoes long periods of rapid change.

### Manufactured datasets

**Fig. 6:**
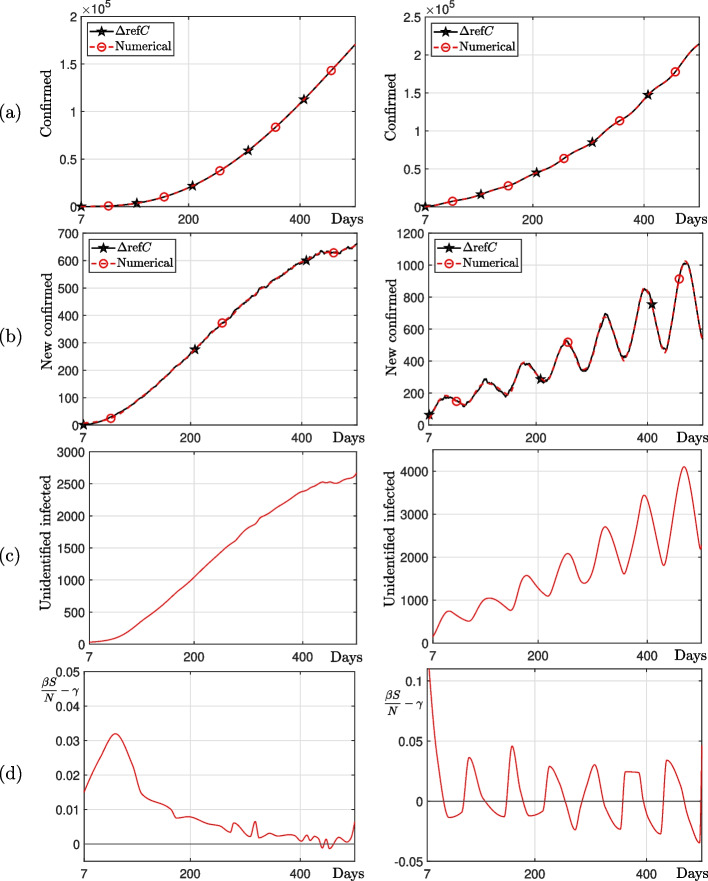
Numerical solutions using the proposed method with the generated datasets. **a**–**c** are number of cumulative confirmed cases, new confirmed cases, and unidentified infected cases, respectively. **d** $\beta S/N-\gamma$. From left to right, the first and second cases

We conducted numerical experiments to demonstrate that the proposed algorithm estimates a time-dependent rate of transmission $\beta (t)$ optimized for tSUC models on various datasets. We generate cumulative confirmed case data based on two new confirmed cases manufactured. The first case was created so that the number of new cases generally increased, including random perturbations, and the second case was created so that the number of new cases increased periodically and gradually, including random perturbations. The parameters used are the total population $N=10^{10}$, $\Delta t=0.1$, $\gamma =1/4$, ${\varvec{\beta }}^0=[1/3,~1/3,~\cdots ,~1/3]$, $U_0^0 = 50$, $lb = [0,~0,~\cdots ,~0,~1]$, $ub = [1,~1,~\cdots ,~1,~\infty ]$, and the number of linear change points $P=30$. Figure 6 shows the numerical results of the generated cumulative confirmed cases. The left column represents the first case and the right column represents the second case. We compare numerical solutions calculated using the estimated $\beta (t)$ with given data and observe that the proposed algorithm estimates the optimal time-dependent transfer rate $\beta (t)$ for various datasets.

### Effect of the number of linear change points *p*

**Fig. 7:**
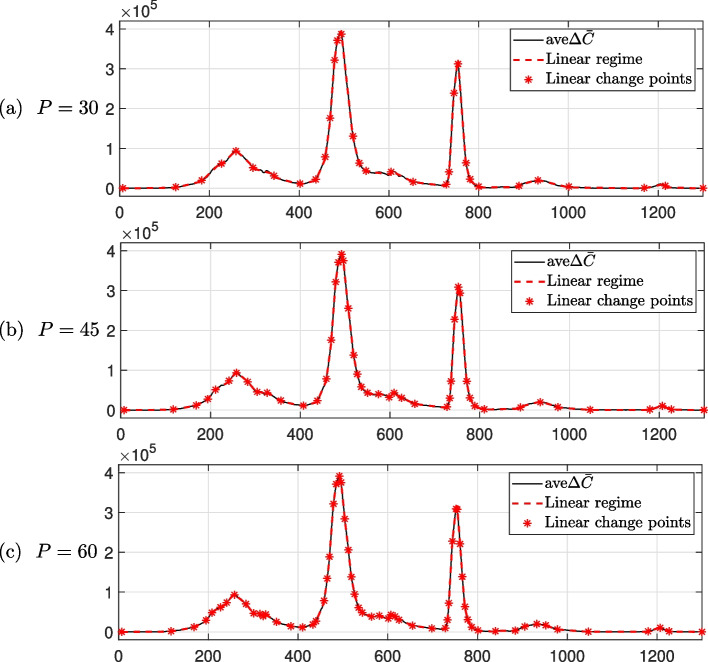
Linear change points identified using the MATLAB function ischange with COVID-19 confirmed data in India

**Fig. 8:**
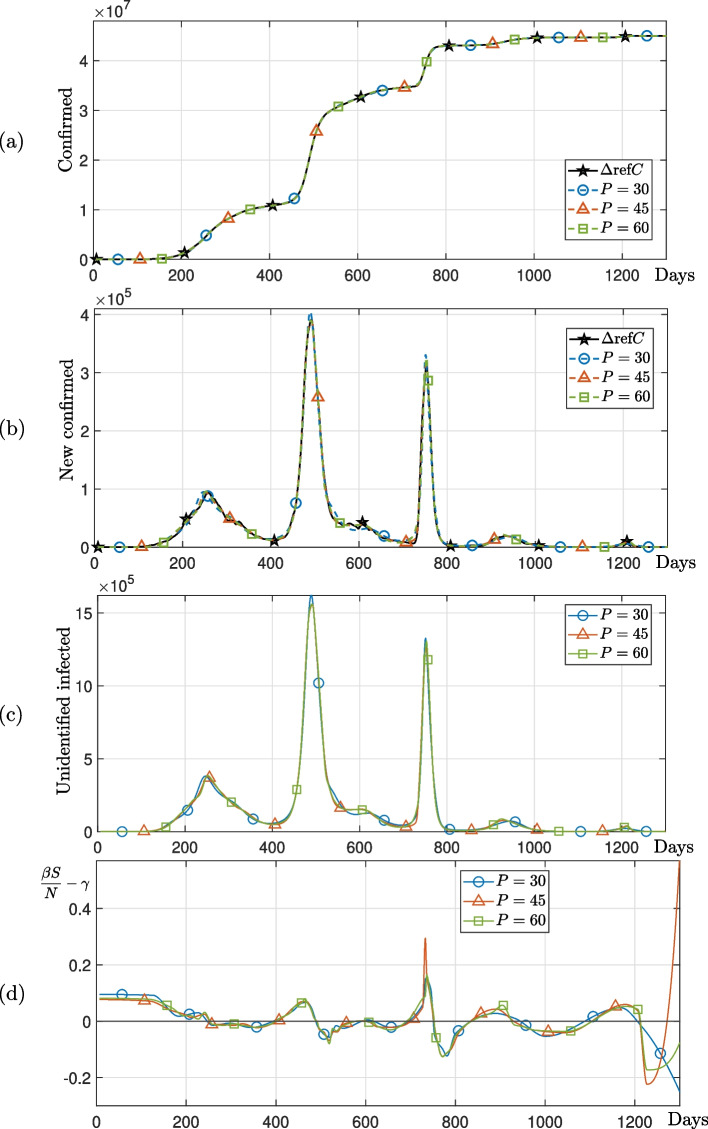
Numerical solutions using the proposed method with COVID-19 confirmed data in India. **a**–**c** are number of cumulative confirmed cases, new confirmed cases, and unidentified infected cases, respectively. **d** $\beta S/N-\gamma$

We consider the effect of the number of linear change points *P*. The parameters used are the total population $N=136\times 10^7$, $\Delta t=0.1$, $\gamma =1/4$, ${\varvec{\beta }}^0=[1/3,~1/3,~\cdots ,~1/3]$, $U_0^0 = 1$, $lb = [0,~0,~\cdots ,~0,~1]$, $ub = [1,~1,~\cdots ,~1,~\infty ]$, and the different number of the linear change points $P=30,~45,~60$. Figure 7 shows the linear change points ${\varvec{t}}=[t_1,~\cdots ,~t_{P+2}]$ found using the MATLAB ischange function with $P=30,~45,~60$ from the confirmed data in India. Then, we obtain the estimated optimal parameters ${\varvec{\beta }}$ and $U_0$ using the Eqs. (4)–(6) and MATLAB function lsqcurvefit. Figure 8 shows that the numerical solutions using Eqs. (4)–(6) with the obtained the estimated optimal parameters. We observed that the cumulative confirmed cases calculated using the optimal parameters from the proposed method closely match the actual cumulative confirmed cases in India. The numerical results show fluctuations in unidentified infections, which provide valuable insights for analyzing asymptomatic infections and diseases with latent periods, such as COVID-19. Unlike the previous method, the results of numerical tests demonstrate that the proposed approach is suitable for explaining the epidemiology of unidentified infections during the three sharp increases in the number of new confirmed cases in India. In addition, the numerical solution shows similar results according to the number of linear change points *P*. This means that more than $P=30$ sufficiently reflects the change in the given COVID-19 confirmed data in India data.

## Conclusion

We proposed the numerical algorithm to obtain the optimal epidemic parameters for the tSUC model. Estimating the parameters of the epidemic model is an important problem in analyzing infectious diseases. The algorithm for estimating the optimal parameters of the epidemic model is structured as follows. First, the data is smoothed through preprocessing. Then, linear change points are calculated based on the smoothed data. Using these calculated linear change points and the tSUC model, the optimal parameters are estimated. We performed numerical experiments to ensure that the proposed algorithm properly estimates the parameters. Numerical experiments showed that the estimated parameters can reproduce the actual COVID-19 epidemiological data of India. In addition, the dynamics of unidentified infected people in India were analyzed using the parameters of the tSUC model estimated using the proposed algorithm. The estimated number of unidentified infected cases using the proposed algorithm is an important result that can serve as evidence supporting the effectiveness of various preventive measures, such as social distancing or wearing masks. In future work, we will develop an appropriate index for the SUC model corresponding to the basic reproduction number $R_0$ of the SIR model, which could be utilized for the analysis of various infectious diseases including COVID-19 where the dynamics of unidentified infected cases are important. In addition, we will consider the time-space-dependent SUC epidemic model, which considers time and space, to analyze the infectious disease including interregional characteristics. Further research will be aimed at enhancing and analyzing these models through the incorporation of additional data and their application to other infectious diseases could significantly broaden public health utility, making it a valuable area for future study [42, 43].

## Data Availability

All data used in this article can be shared upon appropriate request.
